# Some of the More Common Rachitic Deformities

**Published:** 1883-01

**Authors:** V. P. Gibney

**Affiliations:** Professor of Orthopedic Surgery in the New York Polyclinic


ARTICLE II.

### SOME OF THE MORE COMMON RACHITIC DEFORMITIES.

BY V. P. GIBNEY, M. D.

Professor of Orthopedic Surgery in the New York Polyclinic.

The question which the practitioner more frequently than any other propounds to the members of my own specialty is: What shall I do for weak ankles, or bow-legs, or knock-knees, or other deformities known as rachitic? It seemed to me that a short paper on this subject—an answer to this question—might not be out of place.

It shall not be my province to discuss the causes and the mechanism of these deformities. That would consume too much space.

Given, for instance, a case of weak ankles in a child presumably rachitic. What shall be done? What will happen if nothing is done? Will the child grow out of it?

It matters much what the hygienic surroundings of such a child are. If it live in the country, or in a town where densely crowded tenement houses do not prevail—if in commodious quarters with play grounds free from the stench of decaying vegetation; if cleanliness is recognized as one of the cardinal virtues—springs, as a rule, are unnecessary. Of course the degree of deformity must also serve as a guide. The child may be, and generally is, fat and chubby, and is regarded as a large specimen for its age. Treat the rachitis on sound principles, recognizing that for all practical purposes rachitis may be defined as malnutrition. Keep the baby off the feet as much as possible, and let the case be under observation a few months.

Should, however, the case be in a family whose general mode of living and whose hygiene is aught but good, and should the child itself show other signs of poor recuperation, then a support for the ankles can be easily constructed.

In the first place have a good shoe if possible, with a good broad shank and spring heel—immaterial as to whether it be laced or buttoned. Let a smith or shoemaker, under your own instruction, attach by means of rivets a piece of steel to the shank, curved up to the inner ankle, say from a half to three-quarters of an inch in width, according to the strength of the same. To this upright or steel counter have riveted, with a washer for motion, opposite the ankle another spring, narrower and extending to a calf band, to which it can be securely riveted. Any kind of leather or cloth will suit for covering, according to one's taste. Simpler still, a steel shank well arched can be fastened within the shoe, and this will answer admirably for cases not much exaggerated.

A great many weak ankles in children somehow right themselves, and if the tendency should seem contrary to this termination, one will be censurable in not providing against it.

Many cases of flat-foot in young girls and lads date directly from this period.

In very many children brought up in a large city a common deformity—bow-legs—comes on between the end of the first year and the end of the second. Not that some do not appear during the first few months of life, and some even beyond the third year. Yet in the former the treatment is very simple, and any parent even, under a little advice from the physician, can construct such splints or employ such manipulation as will overcome the deformity. The bones are generally susceptible to springing by moderate force. The impression among a large class of practitioners is that children with bow-legs outgrow the bending and ultimately come entirely straight. What foundation is there for such an impression? As a matter of fact, one sees, in this country at least, very few cases of bow-legs in the adult. The foundation is this: many cases are due to a simple relaxation of the ligaments about the knee-joint. These show a wide deviation at the knees, and the curve is a general one, extending from the groin to the malleolus. Such cases nearly always recover spontaneously. Very many have the chief curve in the middle and lower thirds of the leg, and, while the distance from knee to ankle is short, this curve seems very great, and the deformity is quite marked. As this distance increases while the child is "growing out of it" the chord of the arc becomes longer, and when adolescence is attained the curve seems, through the clothing, insignificant. It is really, however, present, and the deviation is just as great. I have examined many such curves, and my statement is based on fact.

This deformity, where it exists in the leg bones, should be corrected by means of springs, and for the physician who is remote from the ordinary appliances well padded splints extending from the inner condyle to the inner side of the heel can be readily employed together with a rubber bandage or adhesive plaster, or rollers even. It will not do the child, as a rule, any harm to keep it off the feet a few weeks or months even. The ordinary bow-leg springs of the shops, though, seem to me very ineffectual. They are attached to shoes, and very little pressure is applied against the convexity of the limbs. They are not worn at night and are thus further objectionable. A steel or leather sole plate with a heel cup attached can be readily constructed by any blacksmith, if other smiths are not in reach. To this heel cup steel springs—the one on the inner side extending to a point over the internal condyle, where a pad is attached; the other on the outer side two inches shorter, and connecting at its upper extremity with the inner band by a narrow steel band passing back of the calf—can be fastened by a rivet and washer opposite the normal tibio-tarsal joint. Then with a roller or a bit of canvas, the limb at its place of deformity can be approximated to the inner spring or bar from ankle to condyles. They need to be worn from four to twelve months.

If the child be over four years of age these will do no good even if worn indefinitely. Resort must then be had to osteotomy, if the deformity be great; otherwise let the case alone.

Knock-knees in rickety subjects are induced by relaxation of the ligaments at the knee—the internal lateral; by atrophy of the external and hypertrophy of the internal condyles; by antero-lateral curvature of the femur, or by hypertrophy upward of the inner head of the tibia.

It is seldom that one finds these conditions separate and distinct. They generally merge one into the other and contribute their respective parts to the production of the deformity.

In very young children the correction can be brought about by forced movements in the opposite direction several times daily. Even in older children, say from two to four years of age, much can be done by this mode of procedure. My friend Dr. Little, of London, advocates a very simple method, figured in his book on *In-Knee Deviation* (London,

1882. p. 115.) A disc of cork from one-half to three-quarters of an inch thick is covered with a layer of cotton-wool and sewed up with silk. This is placed between the internal condyles, and serves as a fulcrum, while each leg is grasped at the ankle by the manipulator's hand and both approximated. Gentleness should characterize the process, and each "sitting" should occupy a half-hour or so. Let it be repeated every three or four hours. The knee must not be allowed to flex. If longer traction is needed on the external lateral ligaments means for securing the feet together will readily suggest themselves to any practitioner. If the case be an intractable one, and seem not to yield any to the mechanical appliances, let the patient be retained under observation from this time until six or eight years of age. Then a supra-condyloid osteotomy (Dr. MacEwen) will as a rule be the best operation.

Pigeon-breast is a not uncommon result of rachitis, and can be overcome when not far advanced by the mother's hand properly directed. Let her several times a day make pressure over the sternum. The child's cries will all the better develop the lungs and assist in restoring the chest walls to normal shape. The hand failing, or the bones being at first too well developed, a spring like a truss-spring can be easily constructed and made comfortable after a few days' use.

What if the deformity is unrelieved?

Just what the ulterior disadvantages may be can only be a matter of speculation. Naturally one would suppose that a barrel-shaped thorax, or pigeon-breasted thorax, would interfere with the proper expansion of the lungs, and when such organs are attacked by disease all the capacity possible would be called into requisition. If the child be a girl, and expects to enter society, a pigeon-breast does not present an attractive figure.

There is another deformity due to rachitis about which many, both in and out of the profession, give themselves needless alarm. I refer to antero-posterior curvature of the spine—rachitic kyphosis. This can very readily be diagnosticated from vertebral ostitis or Pott's disease, inasmuch as it occurs in very young children (under eighteen months of age) and can be overcome by a moderate amount of traction. The deformity always occurs in the dorso-lumbar region, is a general curve very marked as the patient attempts to sit erect. The only treatment needed for this is the prone position, or even the dorsal. If the former, let the mother place the child across her knees, keeping them sufficiently wide apart to allow the sternum to rest on one and the thighs on the other ; then with her hand or hands press upon the convexity of the curve. If in the dorsal decubitus the baby can be balanced on one of the mother's knees, the highest point of the convexity resting on the knee as a fulcrum. If the idea be clearly conveyed to the nurse or mother, a cure will follow in a few months, and no machinery will be necessary, not even a plaster-of-Paris jacket.

Lateral curvature of the spine proceeds from rachitis in this wise : the transverse processes are disturbed in their horizontal relationship by means of faulty position, one facet is mis-shapen a little, and this condition remaining, a rotation of the bodies is induced by the particular vocation in later years, the rachitic changes being the predisposing cause. Hence the importance of proper handling of babies suffering from rickets.

I have occasionally seen subluxation of the sternal ends of the clavicles regarded as rachitic, and many cases of delayed development are undoubtedly attributable to the great disease of mal-nutrition.

